# Increasing the number of available ranks in virus taxonomy from five to ten and adopting the Baltimore classes as taxa at the basal rank

**DOI:** 10.1007/s00705-018-3915-6

**Published:** 2018-06-26

**Authors:** Alexander E. Gorbalenya

**Affiliations:** 1 Department of Medical Microbiology, Leiden University Medical Center, 2300 RC Leiden, The Netherlands; 2 Faculty of Bioengineering and Bioinformatics, Lomonosov Moscow State University, 119899 Moscow, Russia

## Abstract

This opinion article makes a case for increasing the number of ranks used in virus taxonomy from the current five to ten (as are used to classify cellular life forms) and placing the Baltimore classes in the proposed basal rank of domain. These suggestions aim at initiating the process of accommodation of Baltimore classes in virus taxonomy and extension of the virus taxonomy scale to encompass also the most distant relationships.

The International Committee on Taxonomy of Viruses (ICTV) oversees the development of hierarchical virus taxonomy according to the rank structure introduced in 1970 and expanded on several occasions [2]. Currently, it encompasses five ranks, including Order, Family, Subfamily, Genus and Species, in decreasing levels of virus diversity (Virus Code 3.2). These ranks were adopted from those used in the modern version of the Linnaean taxonomy of cellular life forms (virus hosts), which recognizes eight major ranks and may include additional optional ranks. The ranks lacking in virus taxonomy are predominantly above the Order rank (Table 1). Apparently, the limited number of ranks were adopted in virus taxonomy because, at the time, they were sufficient for classifying virus diversity, although a system to embrace the viral world as a whole was already proposed [21]. When the ICTV was founded, only a few dozen viruses from mostly humans, economically important animal and plant hosts and a few bacteria had been identified [29], and there was no indication of the coming grand scale of virus discovery that we are experiencing nowadays (e.g. [4, 12, 28]).

**Table 1:** Rank structure of Virus Taxonomy, currently used and proposed

Rank	Currently used^1^	Proposed^1^
1		Domain
2		Kingdom
3		Phylum
4		Class
5	Order	Order
6		Suborder
7	Family	Family
8	Subfamily	Subfamily
9	Genus	Genus
10	Species	Species

^1^Major ranks are left indented

This contemporary taxonomic structure with its modifications has served virology and virologists for fifty years, when the number of established virus families increased from two to more than one hundred and the number of recognized virus species increased by some two orders of magnitude [2]. Also, the number of ranks that were populated increased from the initial two, genus and family, to the current five, including also species, subfamily, and order, as our knowledge about the natural diversity of viruses and our understanding about the complexity of relations have increased dramatically over these years. (Regretfully, only the introduction and development of the species rank have been extensively documented [26, 27]).

The roots of this increased knowledge and rank expansion could be traced back to the advent of virus genome sequencing and comparative virus genomics, introduced in the end of 1970s and beginning of 1980s [6, 13, 23, 25]. Comparative virus genomics revealed sequence conservations at the family level and above, that had previously been considered unattainable (reviewed at the time in [7, 24]). Many taxa at different ranks, which were established using phenotypic characteristics, were revised, as a result. Now, with the large-scale discovery of viruses in diverse hosts and habitats through genome sequencing, and the increased sophistication of computational methods for the quantification of phylogenetic relationships, it is becoming increasingly evident that the ICTV taxonomy rank structure is insufficient to accommodate properly the emerging scale of virus diversity and the complexity of virus phylogeny. This inconsonance also becomes a practical matter since the ICTV is about to formally recognize the on-going shift from phenotypic- to genomic-based virus taxonomy. (Which it did [3], after the taxonomy proposal underlying this article was submitted).

Below, I list several arguments in favor of adopting an expanded version of the rank structure of the modern Linnaean taxonomy for virus taxonomy and adoption of the Baltimore groupings in the rank of domain, which might be seen as a revival of the idea put forward by Lwoff, Turnier, and Horne [21]. It should be noted that specific examples given below, predominantly from my research and biased for this reason, are NOT taxonomic proposals but are used to illustrate general principles.

## Argument 1

General. The virus host imposes major constraints on virus divergence which effectively links virus and host diversities [15]. Due to the high mutation rate of viruses and the apparent lack of virus-free hosts, it is likely that virus diversity may not be smaller, and is more likely larger than host diversity. Consequently, it could be argued that the number of ranks used to classify viruses should parallel the ranks used to classify hosts, if sufficient virus diversity is available for analysis.

## Argument 2

**Fig. 1:**
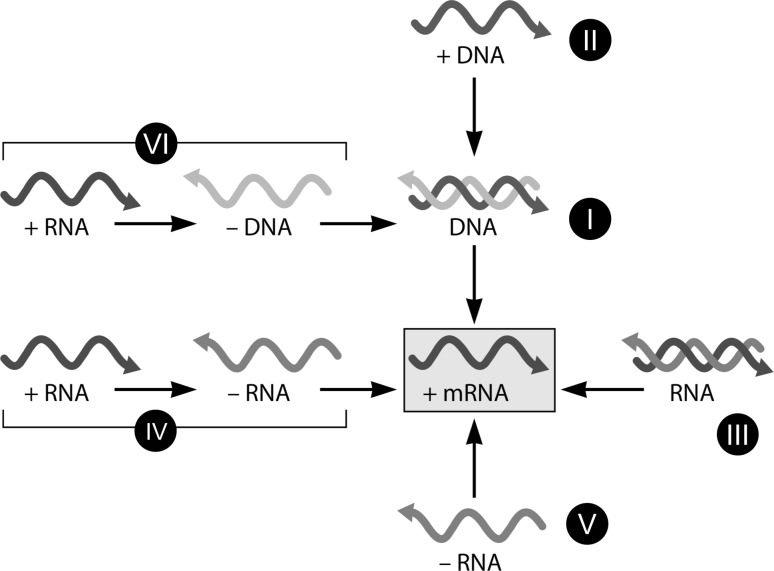
Baltimore classification and its basis. After Fig. 1 of Ref. [5]. (Modified with permission from Flint et al. 2015, Principles of Virology, 4th Edition, Chapter 1, p. 21;©2015 American Society for Microbiology. Used with permission. No further reproduction or distribution is permitted without the prior written permission of American Society for Microbiology.)

Domain and Class. Virologists recognize the Baltimore classification of viruses into six (or seven occasionally) classes (Fig. 1) [5] at its foundation. However, the term “class” itself is not a formally recognized rank in virus taxonomy [14]. This discrepancy is repeatedly highlighted by many virologists, and it calls for action. It would be worth considering recognizing the Baltimore groupings as taxa of the “domain” rather than class rank, which is basal level of the Linnaean taxonomy that encompasses most distant relationships.

Unlike currently recognized taxa, Baltimore classes were established using purely functional considerations concerning genome type and its expression. Its broad albeit informal use in taxonomy is due to overall (perceived) good agreement between these classes and taxa that are recognized phylogenetically. However, this correspondence is not universal, as, for instance, was demonstrated for the dsRNA *Birnaviridae* and the ssRNA + *Permutotetraviridae,* which form an inter-class monophyletic group [9, 30]. There are few other examples of complex relationship between Baltimore classes and phylogenetic groups.

The formal recognition of Baltimore classes at the basal rank of virus taxonomy would open these newly formed taxa, as any other taxa, for oversight and revision by practitioners under the ICTV auspices. Using the conventional taxonomy proposal framework of creating, dissolving, and moving taxa, and coupling it with evolutionary reasoning and public debate, the placement and composition of the original taxa including Baltimore classes could be refined, and a public record of the revision and its reasoning created. The formal recognition of Baltimore classes as taxa at the basal rank of virus taxonomy would also (re-)define the scale of the entire taxonomy, which is informative for defining scales of other newly created ranks. Combined these changes will contribute to the advancement of virus taxonomy and its use in teaching, research, and practical applications.

## Argument 3

Order and above. While the basal rank of virus taxonomy is order, several “super-order” groupings of viruses have been described on phylogenetic grounds. For ssRNA + viruses, they are known as supergroups or superfamilies [7, 8, 24]. For instance, the Picornavirus-like and Alphavirus-like supergroups each include a single order as a subset, *Picornavirales* [20] and *Tymovirales* [1], respectively, as well as many other more distantly related families. In the case of Picornavirus-like supergroup [16, 20], these families, e.g. *Caliciviridae* and *Potyviridae*, share different number of conserved domains and separated by different distances from the *Picornavirales,* indicating that more than a single additional level above order would be required to accommodate them fully in an hierarchical structure.

This example is not an exception, as was shown by our extensive analysis of the order *Nidovirales*, which comprises a distinct supergroup of ssRNA + viruses [8]. This order includes two large monophyletic sets of viruses above the family level (“sub-order”), which are known as small and large nidoviruses, respectively [11]. They are recognized using phylogeny of the most conserved proteins and the presence/absence of the replicative riboexonuclease, ExoN, that serves as a marker domain whose presence correlates with genome size [22]. An extra rank between family and order would reflect better the relationship between the phylogeny and the taxonomy of nidoviruses. An alternative solution would be the introduction of an extra rank above the taxa order and elevation of the current nidovirus order to this rank. Since *Nidovirales* distantly resemble *Astroviridae* and a subset of *Luteoviridae* [11] and all these together have sequence affinity to the Picornavirus-like supergroup [8], further additional ranks could be filled to reflect these relationships.

Clearly, the availability of extra levels above the family rank will facilitate the taxonomy development of other, currently less structured supergroups of ssRNA + viruses of plants and animals, and other classes of viruses, which have highly diverse monophyletic groups, e.g. *Reoviridae*, *Mononegavirales*, etc.

## Argument 4

Family. Using a rigorous method for quantifying the statistical support for clusters and ranks called DEmARC [17], we partitioned the genomic diversity of several RNA virus families into hierarchical classifications. We observed that these classifications included extra level(s) (ranks), whose support was comparable with those of other ranks currently recognized in taxonomy [18]. It could be argued that, at the moment, the extra level(s) in these genetics-based classification of, for example, arteriviruses and filoviruses [19] might have been observed due to the relatively small sampling of viruses in these families (< 700 genomes). However, this explanation seems unlikely for the *Coronaviridae* and, particularly, *Picornaviridae* [17] families, which are amongst the most well sampled groups of viruses studied. In fact, an extra level supported by DEmARC, below the current genus level and called “subgroup” [10], is used by practicing coronavirologists because of its biological relevance. Although the Linneaen taxonomic structure does not offer extra major levels below the family rank, the availability extra levels above the family rank could be used to improve the correspondence between taxonomy and genome-base classification.

## Conclusions

The current taxonomic rank structure of five levels was developed to accommodate an ever-increasing but still relatively limited virus diversity known to us, at a time when virus discovery was mostly an annual event to celebrate. Now viruses are discovered daily if not hourly. The number of known virus species is expected increase from the current thousands to zillions in the future and their classification will be driven by comparative genomics. To accommodate the complexity of phylogenetic relationships apparent within this fast growing diversity, the virus taxonomic rank structure must be adjusted accordingly, as has already become evident from bioinformatics analysis of few better characterized groups of viruses. Using an expanded version of the Linnaean taxonomic structure of ten ranks (eight canonical plus two optional) will contribute to a better description of virus diversity and improve cross-talk between the taxonomies of viruses and their hosts. The number of these ranks could be revisited and, if necessarily, expanded further in the future. Besides the obvious changes to the Virus Code, the formal recognition of the Baltimore classes at the basal level of virus taxonomy could be the first practical steps in this direction.

## References

[1] Adams MJ, Candresse T, Hammond JM, Kreuze J, Martelli GP, Namba S, Pearson MN, Ryu KH, Vaira AM, King AMQ, Adams MJ, Carstens EB, Lefkowitz EJ (2012). Alphaflexiridae. Virus taxonomy ninth report of the international committee on taxonomy of viruses.

[2] Adams MJ, Hendrickson RC, Dempsey DM, Lefkowitz EJ (2015). Tracking the changes in virus taxonomy. Arch Virol.

[3] Adams MJ, Lefkowitz EJ, King AMQ, Harrach B, Harrison RL, Knowles NJ, Kropinski AM, Krupovic M, Kuhn JH, Mushegian AR, Nibert ML, Sabanadzovic S, Sanfacon H, Siddell SG, Simmonds P, Varsani A, Zerbini FM, Orton RJ, Smith DB, Gorbalenya AE, Davison AJ (2017). 50 years of the international committee on taxonomy of viruses: progress and prospects. Arch Virol.

[4] Angly FE, Felts B, Breitbart M, Salamon P, Edwards RA, Carlson C, Chan AM, Haynes M, Kelley S, Liu H, Mahaffy JM, Mueller JE, Nulton J, Olson R, Parsons R, Rayhawk S, Suttle CA, Rohwer F (2006). The marine viromes of four oceanic regions. PLoS Biol.

[5] Baltimore D (1971). Expression of animal virus genomes. Bacteriol Rev.

[6] Fiers W, Contreras R, Duerinck F, Haegeman G, Iserentant D, Merregaert J, Minjou W, Molemans F, Raeymaekers A, Vandenberghe A, Volckaert G, Ysebaert M (1976). Complete nucleotide-sequence of bacteriophage Ms2-Rna—primary and secondary structure of replicase gene. Nature.

[7] Goldbach RW (1986). Molecular evolution of plant RNA viruses. Annu Rev Phytopathol.

[8] Gorbalenya AE, Gibbs AJ, Calisher CH, Garcia-Arenal F (1995). Origin of RNA viral genomes; approaching the problem by comparative sequence analysis. Molecular basis of virus evolution.

[9] Gorbalenya AE, Pringle FM, Zeddam JL, Luke BT, Cameron CE, Kalmakoff J, Hanzlik TN, Gordon KH, Ward VK (2002). The palm subdomain-based active site is internally permuted in viral RNA-dependent RNA polymerases of an ancient lineage. J Mol Biol.

[10] Gorbalenya AE, Snijder EJ, Spaan WJ (2004). Severe acute respiratory syndrome coronavirus phylogeny: toward consensus. J Virol.

[11] Gorbalenya AE, Enjuanes L, Ziebuhr J, Snijder EJ (2006). Nidovirales: evolving the largest RNA virus genome. Virus Res.

[12] Hatfull GF, Hunters SEAP, My K-NRITH, Educ PHIR (2012). Complete genome sequences of 138 mycobacteriophages. J Virol.

[13] Kamer G, Argos P (1984). Primary structural comparison of Rna-dependent polymerases from plant, animal and bacterial-viruses. Nucleic Acids Res.

[14] King AMQ, Adams MJ, Carstens EB, Lefkowitz EJ (2012). Virus taxonomy, ninth report of the international committee on taxonomy of viruses.

[15] Koonin EV, Gorbalenya AE (1989). Evolution of RNA genomes—does the high mutation-rate necessitate high-rate of evolution of viral-proteins?. J Mol Evol.

[16] Koonin EV, Wolf YI, Nagasaki K, Dolja VV (2008). The big bang of picorna-like virus evolution antedates the radiation of eukaryotic supergroups. Nat Rev Microbiol.

[17] Lauber C, Gorbalenya AE (2012). Partitioning the genetic diversity of a virus family: approach and evaluation through a case study of picornaviruses. J Virol.

[18] Lauber C, Gorbalenya AE (2012). Toward genetics-based virus taxonomy: comparative analysis of a genetics-based classification and the taxonomy of picornaviruses. J Virol.

[19] Lauber C, Gorbalenya AE (2012). Genetics-based classification of filoviruses calls for expanded sampling of genomic sequences. Viruses Basel.

[20] Le Gall O, Christian P, Fauquet CM, King AMQ, Knowles NJ, Nakashima N, Stanway G, Gorbalenya AE (2008). Picornavirales, a proposed order of positive-sense single-stranded RNA viruses with a pseudo-*T* = 3 virion architecture. Arch Virol.

[21] Lwoff A, Tournier P, Horne R (1962). System of viruses. Cold Spring Harb Sym.

[22] Nga PT, Parquet MD, Lauber C, Parida M, Nabeshima T, Yu FX, Thuy NT, Inoue S, Ito T, Okamoto K, Ichinose A, Snijder EJ, Morita K, Gorbalenya AE (2011). Discovery of the first insect nidovirus, a missing evolutionary link in the emergence of the largest RNA virus genomes. PLoS Pathog.

[23] Sanger F, Coulson AR, Friedmann T, Air GM, Barrell BG, Brown NL, Fiddes JC, Hutchison CA, Slocombe PM, Smith M (1978). Nucleotide-sequence of bacteriophage-Phi-X174. J Mol Biol.

[24] Strauss JH, Strauss EG (1988). Evolution of RNA viruses. Annu Rev Microbiol.

[25] Toh H, Hayashida H, Miyata T (1983). Sequence homology between retroviral reverse transcriptase and putative polymerases of hepatitis B virus and cauliflower mosaic virus. Nature (Lond).

[26] Van Regenmortel MH, Maniloff J, Calisher C (1991). The concept of virus species. Arch Virol.

[27] Van Regenmortel MHV (2018). The species problem in virology. Adv Virus Res.

[28] Webster CL, Longdon B, Lewis SH, Obbard DJ (2016). Twenty-five new viruses associated with the drosophilidae (Diptera). Evol Bioinform Online.

[29] Wildy P (1971). Classification and nomenclature of viruses. First report of the international committee on nomenclature of viruses.

[30] Zeddam JL, Gordon KHJ, Lauber C, Alves CAF, Luke BT, Hanzlik TN, Ward VK, Gorbalenya AE (2010). Euprosterna elaeasa virus genome sequence and evolution of the Tetraviridae family: emergence of bipartite genomes and conservation of the VPg signal with the dsRNA Birnaviridae family. Virology.

